# Bio-Inspired Motion Emulation for Social Robots: A Real-Time Trajectory Generation and Control Approach

**DOI:** 10.3390/biomimetics9090557

**Published:** 2024-09-15

**Authors:** Marvin H. Cheng, Po-Lin Huang, Hao-Chuan Chu

**Affiliations:** 1 Department of Mechanical, Material & Aerospace Engineering, West Virginia University, Morgantown, WV 26505, USA; 2 Department of Power Mechanical Engineering, National Tsing-Hua University, Hsinchu 300, Taiwan; s2522238@gmail.com (P.-L.H.); okbe32220205@gmail.com (H.-C.C.)

**Keywords:** motion recognition, sensor fusion, humanoid robotic system

## Abstract

Assistive robotic platforms have recently gained popularity in various healthcare applications, and their use has expanded to social settings such as education, tourism, and manufacturing. These social robots, often in the form of bio-inspired humanoid systems, provide significant psychological and physiological benefits through one-on-one interactions. To optimize the interaction between social robotic platforms and humans, it is crucial for these robots to identify and mimic human motions in real time. This research presents a motion prediction model developed using convolutional neural networks (CNNs) to efficiently determine the type of motions at the initial state. Once identified, the corresponding reactions of the robots are executed by moving their joints along specific trajectories derived through temporal alignment and stored in a pre-selected motion library. In this study, we developed a multi-axial robotic arm integrated with a motion identification model to interact with humans by emulating their movements. The robotic arm follows pre-selected trajectories for corresponding interactions, which are generated based on identified human motions. To address the nonlinearities and cross-coupled dynamics of the robotic system, we applied a control strategy for precise motion tracking. This integrated system ensures that the robotic arm can achieve adequate controlled outcomes, thus validating the feasibility of such an interactive robotic system in providing effective bio-inspired motion emulation.

## 1. Introduction

As society continues to evolve, social robots are playing an increasingly crucial role in enhancing human life across various sectors. From healthcare and education to industrial assistance, these advanced machines have the power to revolutionize our daily lives. By mimicking natural human interactions, social robots provide companionship, support, and efficiency in a wide range of tasks, from personal care to collaborative work [1,2,3]. The rapid advancements in artificial intelligence (AI) and humanoid robotics have significantly contributed to the development of social robots [4]. These innovations enable these machines to recognize, interpret, and replicate human behaviors with remarkable accuracy and responsiveness [5]. This has led to the creation of assistive robotic platforms, also known as exoskeleton devices, specifically designed for the healthcare market.

The development of social robots has been revolutionized by AI technologies such as machine learning and convolutional neural networks (CNNs) [6]. These advancements have enabled social robots to process vast amounts of data, learn from human interactions, and improve their ability to perform complex tasks [7]. Humanoid robots, designed to mimic human form and movements, provide an intuitive interface for interaction, making them more acceptable and effective in social contexts [8]. These technological advancements are crucial for developing robots that can seamlessly integrate into human environments and perform tasks with high precision and adaptability. Over the past decade, researchers have intensively explored various applications of social robots. Assistive exoskeletons, for example, have been developed to aid stroke patients in their recovery and to help seniors with daily activities. Ensuring the safety and efficiency of these robots is crucial and can be achieved by adopting trajectories that mimic common user movements. This approach helps prevent potential injuries caused by unusual movements or sudden loss of force [9,10]. In educational settings, social robots have been developed to provide personalized learning experiences and assist in classroom management. These robots can engage students through interactive lessons and activities, enhancing the overall learning environment and fostering better educational outcomes [11]. As social robots continue to evolve, they have the potential to transform our lives in profound ways, making them an essential component of modern society. 

Recent studies have highlighted the potential of AI-driven motion recognition systems in enhancing the interaction capabilities of robots. In this research, a robotic platform featuring two five-degree-of-freedom robotic arms and a depth camera, designed to mimic a human upper body, was developed to investigate human–robot interaction. CNNs and path planning techniques were utilized to accurately predict and replicate human motions by processing time-series data and converting it into meaningful motion trajectories. Additionally, adaptive robust control (ARC) strategies [12] and a linear controller were implemented to compensate for nonlinear factors in robotic systems and improve steady-state operation, ensuring precise tracking and execution of movements. 

In the experimental setup, a depth camera and a regular camcorder were used for motion capture, leveraging their extensive programming resources and high accuracy. These devices were integrated into the robotic platform to enable direct visualization and recognition of human motions. The captured image and depth data were processed using a skeletal recognition library to derive joint angular information. This information was then processed by the developed motion prediction model utilizing CNNs to interpret angle-to-angle representations of joint movements, allowing for accurate prediction and replication of human actions. 

To generate accurate and safe trajectories for the robotic platform, actual trajectories of targeted motions collected from various users were used to develop reference trajectories. Previous studies have shown that motion profiles are generally similar among healthy adults, although small deviations and varying durations are common even for repeated motions by the same person [13]. Though this study focused on the motion prediction for a single user, the motion prediction model is capable of providing distinct predictions for individual motions. Once the motion type was predicted, the corresponding reference trajectories were generated. To achieve this, temporal alignment of time series, a method investigated by various research groups, was adopted to align collected profiles onto a single timeframe, enabling the derivation of reference trajectories. Dynamic time warping (DTW) was used for spatiotemporal alignment among multiple time series [14], with the generated reference trajectories scaled from 0% to 100%. This approach allows the output timeframe to be adjusted to the required duration, ensuring adequate path synthesis for the robotic platform. 

When comparing the results of this study with those from previous research, the proposed method, combining CNN modeling with a DTW-path planning approach, demonstrates substantial advancements over earlier methodologies. This superiority is primarily due to several factors, including enhanced accuracy, scalability, and the ability to handle complex motion patterns. 

Traditional path planning for robotic systems has often relied on deterministic algorithms or classical machine learning models. For example, methods like Dijkstra’s algorithm, A* algorithms, and recursive inverse kinematics have been commonly used for spatial pathfinding in collaborative robotic systems, as seen in the works by Cheng et al. [15] and Sohn et al. [16]. While these methods are effective for basic end effector movement tasks for robotic arms, these methods often struggle with dynamic environments or complex motion patterns. In contrast, the proposed approach, which integrates CNN-based modeling and DTW synthesis of movement trajectories, leverages deep learning to predict and understand complex trajectories with higher precision. By using convolutional layers to extract spatial features from motion data, our method achieves a more nuanced understanding of the task. The proposed control strategy also provided better tracking and synchronization accuracy. 

Scalability and flexibility are critical for path planning in assistive devices, yet previous methods often lacked these qualities, especially when applied to diverse motion patterns across different users [17,18]. The proposed approach, however, allows for the scaling of reference trajectories based on individual user characteristics, such as body size and motion duration. This adaptability represents a significant improvement, enabling the system to cater to a broader population and a wider range of applications.

Moreover, previous research frequently overlooked nonlinear factors affecting robotic motion, such as varying inertia, inconsistent friction, and dead zones in motors. For instance, in Liu et al. [19], while the focus was on optimizing the path planning algorithm, the impact of these nonlinearities was not adequately addressed. In contrast, the proposed approach integrates these nonlinearities directly into the control model using an ARC mechanism, which compensates for both structured and unstructured uncertainties. This integration results in more reliable and stable performance, particularly in tasks requiring fine motor control. Additionally, the combined use of ARC and linear controllers in the proposed control strategy effectively addresses both nonlinearities and steady-state tracking performance. The ARC controller compensates for nonlinear factors such as dead zones and actuator saturation, while the linear controller ensures smoother tracking during steady-state conditions. This integrated approach optimizes the tracking performance of the robotic platform for various motions.

In summary, the proposed method based on prediction and the temporal path planning approach in this study demonstrates significant improvements in handling complex, real-time robotic tasks by incorporating advanced machine learning techniques, robust adaptive control mechanisms, and a scalable framework. These enhancements make it superior to previous research methods, offering a more versatile and reliable solution for motion planning in robotic systems.

The structure of this paper is as follows: Section 2 covers the motion capture setup and data processing techniques utilized in this study. Section 3 discusses the experimental setup of the robotic platform. Section 4 elaborates on the motion recognition system and the development of the CNN-based angle-to-angle representation. Section 5 focuses on the design and implementation of the integrated ARC and linear control strategy. This section also presents the simulated results and compares the tracking performance of the proposed controllers. It also discusses the strategies of the integrated control scheme. Section 6 explains how the robotic platform mimics human motion and addresses motion duration adjustments. Finally, Section 7 concludes the paper with a summary of findings and future research directions.

## 2. Computer Vision for Human Motion Acquisition

Recently, humanoid robots have gained increasing popularity. These robots not only provide a sense of comfort and familiarity to humans but can also imitate human actions, performing tasks that users typically do themselves. For a social or humanoid robot to effectively mimic human motion, it must accurately understand human biomechanics and the trajectories that individual joints follow during movement. This requires sophisticated motion acquisition and processing systems that can capture and analyze the nuances of human motion in real time. By integrating advanced sensors and algorithms, these robots can learn and replicate complex human activities, enhancing their utility in various applications such as personal assistance, healthcare, and entertainment. 

### 2.1. Motion Acquisition Devices

Over the past decade, numerous motion acquisition techniques have been investigated for capturing body movements. When choosing a suitable motion acquisition method for humanoid or social robots, several factors must be considered, including the integration of sensors into the robotic system. The cost of these systems varies widely depending on the technology used. To balance cost and accuracy, this study tested three acquisition methods:

1. Depth Camera: Depth cameras enable the simultaneous acquisition of individual joint movements in three-dimensional space with minimal limitations. However, they tend to be more expensive than regular camcorders. Both camcorders and depth cameras offer the advantage of non-contact acquisition but require operation within a designated space. Additionally, measurements from these cameras can be distorted by environmental conditions.
2. Standard RGB Camcorder: Camcorders, being a mature technology, are widely integrated into almost all laptop computers, making them a cost-effective option for motion acquisition using existing devices. However, like depth cameras, they need to be used within a designated space and can also be affected by environmental factors.
3. Data Fusion System Combining Low-Cost Wearable Gyroscopic Sensors and Accelerometers: Accelerometers and gyroscopic sensors, which are directly attached to users, can capture motion regardless of the surrounding environment. These attached sensors can be easily integrated into an exoskeleton robotic platform as mechatronic components. They are typically low-cost, easily replaceable, and beneficial for their integration flexibility. However, they have limitations in measuring certain types of motion due to mechanical constraints. This type of sensor requires more preparation time before motion acquisition can begin.

Each method has its advantages and limitations. Depth cameras provide detailed 3D motion data but at a higher cost. Camcorders are more affordable and widely accessible but only capture 2D projections and can be affected by environmental conditions. Sensor fusion systems offer flexibility and low cost but require physical attachment to the user and may face mechanical constraints.

These sensors are deployed based on specific scenarios where humanoid and social robots operate. Modern robots are typically equipped with LiDARs or cameras to perceive their surroundings. Cameras, in some cases, can directly facilitate motion acquisition. However, when cameras alone cannot suffice for capturing motion, additional wearable sensors can fill this gap, ensuring that the robots can accurately capture and replicate human movements in various environments and tasks. This dual approach enhances the versatility and reliability of robotic systems in interacting with and assisting humans.

### 2.2. Motion Acquisition Process

To adequately plan the movement trajectories of a robotic platform for human-like motions, the control system must include the trajectories of involved joints, particularly for the arms. To enable a social robot to mimic human motions, a library of reference trajectories needs to be compiled in advance. In this study, the robot platform was programmed to respond to three pre-selected self-similar motions to ensure consistent performance of human-like movements. Self-similar motions were targeted to guarantee that the robot could always perform actions with similar trajectories, enhancing the human-like quality of its movement. However, not all motions exhibit self-similarity. Therefore, this study focused on two specific motions: object lifting and right arm raising. The motions were selected to assist human workers in a warehouse environment. In this setting, it is essential for human workers to recognize robots as trustworthy partners rather than merely another piece of installed equipment. These motions were chosen because they are common in everyday interactions, making their recognition and replication crucial for the robot’s functionality. Once a motion type was identified, the robot mechanism responded accordingly, using baseline trajectories designed for these motions as reference motions for the experimental robotic platform.

**Figure 1:**
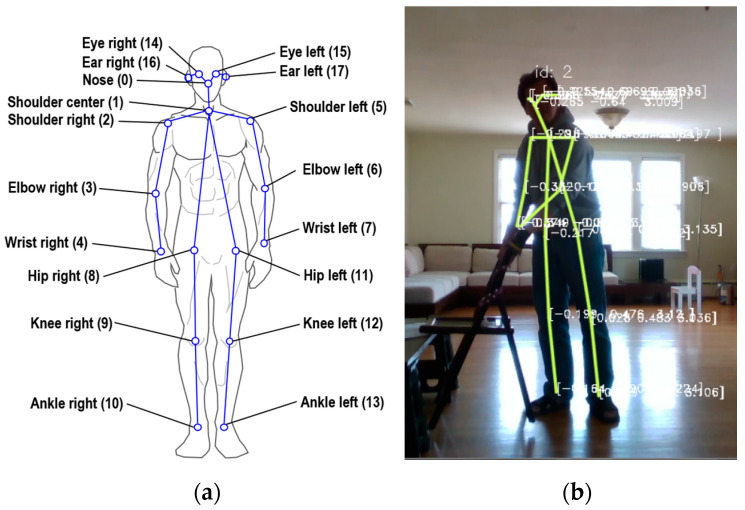
(**a**) Recordable joints and the corresponding locations for human motions using the Cubmos library. (**b**) Image of the moving object motion captured with an Intel RealSense D435 camera and processed using the Cubemos framework in C#.

This study employed two different depth cameras for motion acquisition: a Kinect Xbox 360 depth camera manufactured by Microsoft and a RealSense D435 made by Intel. The Kinect depth camera was chosen for its extensive programming library resources, and a motion acquisition interface was developed using the Microsoft Kinect SDK package v1.8. The motion acquisition rate for the Kinect was set to 25 frames per second. The Intel RealSense D435 was selected for its advanced capabilities, and the motion acquisition interface for this camera was developed using the Cubemos library. To ensure effective capture, the depth camera was placed 3 m in front of each test subject, whose heights ranged from 160 cm to 190 cm, as illustrated in Figure 1a. Upon the motion being recorded, the positions of individual joints in 3D space were captured, and joint angular information was subsequently derived from this spatial data. However, a major drawback of this method is that optical signals can be disrupted, compromising accuracy. The motion data was processed using an AMD Ryzen 7 4700U processor. Each test subject completed 50 trials of each motion. During these trials, the positions of shoulder, elbow, and wrist joints for both arms were recorded. These recorded positions were then converted to the angular movements of the joints of the shoulder and elbow joints. Figure 1b demonstrates the joints involved in the motion acquisition process. For the selected motions, only the time series data of the arm joints were recorded. A modeling method developed by Cheng et al. [13] was employed to determine the motion identification model. The model was subsequently used to identify the ongoing motion and determine the corresponding trajectories for the robotic platform.

Once established, the model can predict the type of ongoing motion, enabling the generation of joint trajectories tailored to these motions. This capability is valuable in healthcare applications, where it can assist patients by providing personalized support based on their movements. Additionally, in various workspaces, such as manufacturing or rehabilitation settings, this predictive ability can enhance efficiency and safety by automating tasks or providing real-time assistance based on observed motions.

This study specifically focuses on motions that can be performed by a single arm. The decision to concentrate on single-arm movements is motivated by the relative simplicity in processing and modeling these motions. Single-arm actions involve fewer degrees of freedom compared to tasks requiring the coordination of both arms or multiple joints, which in turn reduces the computational complexity.

Modeling and controlling movements that engage both arms or involve multiple joints require significantly more intensive calculations and longer processing times. These factors would demand advanced modeling techniques and computational resources, making them outside the scope of this study. By focusing on single-arm motions, the research aims to develop more efficient and streamlined models, allowing for a more targeted exploration of the proposed methods.

### 2.3. Prediction Model of Human Motions

Once the motion data acquired from the depth camera are formatted appropriately, the next step involves immediate identification of the corresponding types of motions. Various pattern recognition techniques have been proposed, including Markov chain [20,21], temporal alignment [22], and other methods. Recent advancements in deep learning models have shown outstanding performance in action recognition [23]. The prediction model can also be used to trigger the robotic platform to react to certain selected motions. This section introduces a motion prediction model based on a deep learning approach.

One efficient approach for recognizing arm motions involves utilizing convolutional neural networks (CNNs) with dynamic motions captured by motion acquisition devices. Instead of relying on time-series information, representing the motion information of involved joints through angle-to-angle data has been proven effective and intuitive. This method simplifies the required calculation by transforming individual time-based data into spatial 3D representations. The adoption of angle-to-angle representation offers several advantages. A single portrait can demonstrate profiles with unique characteristics, making it straightforward to distinguish different motions when the positions of multiple joints are presented geometrically. Considering these factors, 3D arm movements can be processed using 2D calculations by projecting a specific combination of joints onto 2D planes. This can dramatically reduce the required calculation using the neural network.

**Figure 2:**
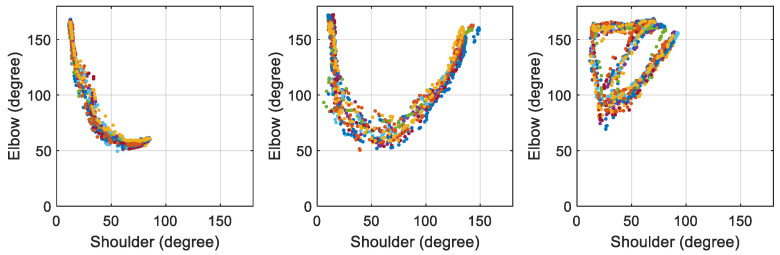
Angle-to-angle plots of three selected motions: drinking water, raising right hand, and object lifting (The different colors represent 10 trials).

Figure 2 illustrates the angle-to-angle representation of the three selected motions collected from a test subject. The unique characteristics of these motion profiles are evident. Even when the same motion is performed at different times, the major features remain the same, allowing for differentiation from other motions. With enough samples collected, a prediction model can be established based on the profiles of the selected motions.

The process of using CNNs for motion recognition follows a similar approach to that of regular image recognition. However, before applying CNNs, a preprocessing step is necessary to convert individual time-series information into angle-to-angle data. The transformed data is then utilized for both modeling and prediction purposes. In this study, once the motions were captured by motion sensors, the movements of the elbow and shoulder joints were selected for transformation into angle-to-angle representations.

**Figure 3:**
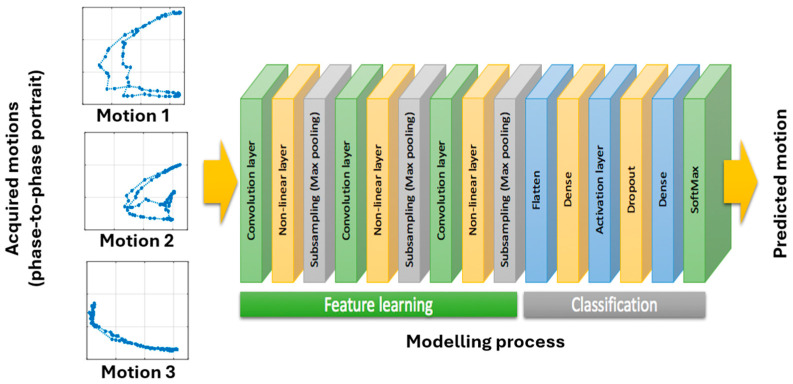
Training process using the framework of CNNs.

To ensure consistency, each set of angle-to-angle data was standardized to a size of 256 × 256 points. To gather a sufficient number of samples for the training process, each motion was recorded 200 to 400 rounds from the test subject. After gathering all the samples, the training process began. This method leverages the unique characteristics of angle-to-angle representations to facilitate accurate and intuitive motion recognition. Figure 3 illustrates the angle-to-angle representation of the three selected motions collected from a test subject, highlighting the distinct features that enable the CNN model to differentiate between different motions effectively. In these figures, 10 trials of each individual motion are displayed. It is evident that the selected motions are consistent and self-similar across all trials performed by the same person.

The training process using CNNs followed a standardized approach, as illustrated in Figure 3 [6]. The angle-to-angle representation served as inputs for the entire process. Three convolution layers were used to extract the major features from the collected motions. To enhance motion recognition, 3 × 3 filters in the convolution layers were selected based on the profiles of individual motions. After each convolution layer, a nonlinear activation function identified distinct features in each hidden layer. A subsampling (max pooling) layer was utilized to reduce the resolution of the features from layer to layer, making them more robust against noise and distortion. The total number of layers in the training process was 15. Upon completion, the classifier generated over four million neurons for the targeted motions. The resulting model was then stored and used to detect ongoing motions. 

In this study, data collected from a single test subject were used for the training process, totaling 162 datasets. An additional 32 datasets were used for verification. Using a Ryzen 7 4700U CPU, the training time for a single model was less than 300 s. The verification and prediction time for each motion was less than 0.5 s. The derived model, utilizing the CNNs method, achieved a prediction success rate of over 90% for the three selected motions when processing just one-third of the motion cycle. Additionally, the model consistently provided correct predictions to indicate that the performed motion was not among the three selected. 

To make the prediction model practical for the robotic platform to react to specific motions, it needs to predict the intended movement at the beginning of a motion rather than waiting for a motion to be completed. In this study, the robotic platform repeated the predicted motion immediately following the prediction. Once the motion was identified, the corresponding joint trajectories were planned. An important objective of using deep learning in robotic platforms is to estimate and generate complete movement trajectories from partial information. This allows the robot to react promptly, providing necessary assistance in healthcare or industrial applications. Therefore, it is critical to determine the minimal portion of a motion required for accurate prediction. This section investigates modeling with partial motion data to ensure timely motion prediction, enabling the humanoid robot platform to deliver the required assistance efficiently.

Previous studies also recommended capturing at least one-third of the total motion to ensure accurate motion prediction with a successful rate greater than 95% [13]. For the path planning of the robotic platform, the robot’s movements were programmed to respond to specific human motions. Therefore, trajectories of individual joints were planned based on motion data after one-third of the motion cycle was performed by the user. Table 1 presents the predicted results generated by the proposed model using the selected motions.

### 2.4. Validation of Prediction Model

To verify the prediction results obtained from CNNs in this study, we employed the *k*-fold cross-validation method. This method is particularly effective for assessing the performance of machine learning models for CNNs, as it provides a robust evaluation of the model’s ability to generalize across different subsets of the data. The entire dataset was divided into *k* subsets, known as folds. The value of *k* is typically chosen based on the size and variability of the data, with common values being 5 or 10. For this study, we selected *k* = 6 to achieve a balance between computational efficiency and a comprehensive evaluation. 

During the training and validation phase, the CNN model was trained on *k* − 1 of these folds, while the remaining fold was used for validation. This process was repeated *k* times, ensuring that each fold served as the validation set once. By averaging the model’s performance across all folds, we obtained a more accurate and reliable estimate of its predictive accuracy. Throughout each iteration, key performance metrics such as accuracy, precision, recall, and *F*1-score were calculated for the validation set. These metrics were then averaged across all folds, providing a comprehensive assessment of the model’s overall performance.

The *k*-fold validation process also offered valuable insights into the model’s strengths and weaknesses. These insights guided adjustments to hyperparameters, architecture, or training strategies, allowing us to address any observed issues such as overfitting or underfitting by refining the CNN model’s configuration. Following the completion of cross-validation, the CNN model was retrained on the entire dataset using the optimized parameters identified during the validation process. This final model was then used to make predictions on unseen data, ensuring that it was both well-tuned and capable of generalizing effectively.

To further enhance the robustness of the model, the *k*-fold validation process was repeated multiple times with different random seeds for data splitting. This repetition reduced the impact of any particular data split on the final results, reinforcing the consistency and reliability of the CNN’s predictions.

By utilizing *k*-fold cross-validation, this study rigorously verified the CNN model’s prediction results, which are 99.6%. The approach ensured that the model performed well not only on the training data but also on new, unseen data, thereby increasing the reliability and applicability of the model in practical scenarios.

## 3. Configuration of the Robotic Platform

**Figure 4:**
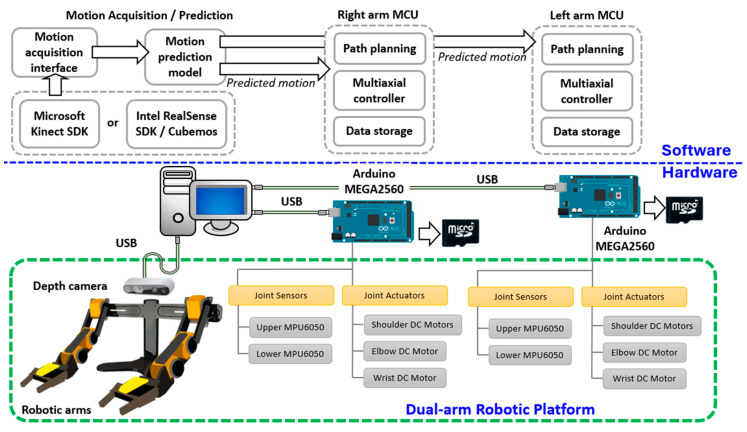
System configuration of the dual-arm robotic platform and the corresponding motion acquisition and control subsystems.

One crucial function of a humanoid robot is to respond to specific human actions. To achieve this, the robotic platform must identify ongoing human motions and react accordingly. This requires the robot’s processing unit to determine its movements based on the types of human motions detected. This goal necessitates the integration of machine vision, human motion modeling, path planning, and control strategies for a multi-axial robotic mechanism. The proposed configuration for such a system in this study is illustrated as Figure 4.

The hardware of the experimental platform was divided into two main components: the motion acquisition/prediction component and the robotic mechanism. The motion acquisition/prediction component was responsible for capturing human movements and determining the motion types. This subsystem integrated an Intel RealSense D435 depth camera and a processing computer. The robotic mechanism included two OWI robotic arms, position sensors, and microcontroller units (MCUs). The predicted motion type from the motion acquisition component was sent to the MCUs of the individual robotic arms to select the corresponding joint trajectories.

With this experimental setup, the robotic platform first predicted the type of ongoing human motions. Once the motion type was determined, the robotic platform performed the corresponding movement in response to the detected human motions. These specific motions for this study were as follows:

(1) Moving an object from a lower position to a higher position.
(2) Raising the right hand.
(3) Drinking a cup of water.

### 3.1. Mechanism of Robotic Platform

In this study, a dual-arm robotic platform mimicking the upper body of a humanoid robot was developed using two robotic arms. The dual-arm mechanism in this study was optimized by using off-the-shelf, battery-powered robotic arms, each with 5-DOF to mimic the natural motion of a human arm. These arms were carefully balanced on a supporting frame to ensure stability and accuracy in motion replication. The 5-DOF configuration was selected to provide sufficient flexibility while keeping the system efficient, with each joint corresponding to a real human arm’s movement, allowing for precise control and operation. This approach streamlined the integration process, leveraging readily available hardware for robust performance. A depth camera was mounted on the upper central section of the mechanism to capture both human motions and the movements of surrounding objects. The mechanism is illustrated in Figure 4. Each arm mechanism in this robotic system included two gyro sensors, two motor driver modules, and a control unit. The shoulder and elbow joints were controlled by a synthesized controller that accounted for cross-coupled dynamics between the included limbs. The trajectories for individual joints were sent by a host controller and stored locally in a SD card.

In each arm’s movement, motor speeds at the shoulder and elbow joints were directly related to their applied voltages. MCUs used for these robotic arms produced pulse-width modulated signals as motor controls. These signals required amplification by H-bridge drivers, specifically L298N modules, enabling smooth joint movement. Accelerometer and gyroscope data from MPU6050 sensors provided real-time feedback on the arm’s orientation and speed at both joints. Individual Arduino Mega2560 MCUs were responsible for managing data collection, sensor integration, executing control algorithms, communication between arms, and saving information to storage mediums. Each MCU employed an I2C interface with MPU6050 sensors for instantaneous motion tracking. The program incorporated pre-loaded joint trajectory data from a text file located on the SD card corresponding to chosen movements to formulate necessary control signals. For storing detailed movement information and facilitating post-processing, the same integrated SD card was used, with the output transmitted via USB connections back to the host computer for examination or presentation purposes.

To simulate real-life conditions in which objects were manipulated, additional external forces can be introduced into this experimental robotic platform. The overall control system was designed to handle higher-frequency operations; it can process functions at over 200 Hz during single-axis movements that include file management tasks and complex sensor integration processes. However, the operational frequency of the MCUs in charge of controlling movement was deliberately limited to a steady 100 Hz. This conservative sampling rate ensured that adequate time wasallocated for executing control algorithms, which are crucial for accurate and stable joint positioning. 

To interact with the users, the dual-arm robotic platform was positioned three meters away from the user, ensuring that the depth camera had sufficient distance to accurately detect human motion. As illustrated in Figure 4, the motion prediction model was triggered once the depth camera detected a human. If the performed motion matched one of the three selected motions, the corresponding reference motion type and necessary parameters were sent to the MCUs of the individual arms. The reference motion trajectories, stored in pre-saved files, were then loaded. These trajectories were adjusted based on the acquired human motion to ensure precise alignment. Finally, the adjusted trajectories were fed to the controllers to compensate for the tracking performance. 

### 3.2. Modeling of Joint Actuators

Developing a controller that can effectively compensate for the joint movements of the robotic platform requires creating theoretical models representing individual joint dynamics. The mechanical components in question are driven by independent DC motors and manifest both linear and nonlinear characteristics in their responses. To simplify the modeling effort, the linear dynamics of each joint were analyzed to compensate for angular movements. The initial step involved determining essential parameters from a motor’s response to step inputs to establish transfer functions that characterize the linear aspects of these joints. 

**Figure 5:**
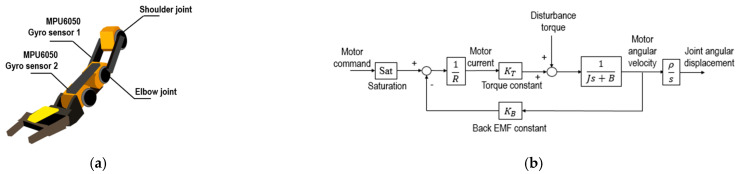
(**a**) Locations of DC motors and sensors. (**b**) Block diagram of a single DC motor used for joint angular movement.

Figure 5a illustrates where the DC motors and sensors were located. While individual discrepancies in coefficients due to hardware-induced nonlinearities may occur, all motors driving the robotic structure were homogeneously DC-powered. Nonetheless, variations in inertia among different limbs—the upper and lower ones—emerged from their orientation relative to the mechanism’s pose and which motor was active. These dynamic fluctuations were integrated into the modeling phase, allowing for a refined discretization of linear equations within each joint’s controller design framework. A simple first-order transfer function was implemented as an approximation for predicting the motion of each joint. It is

(1) $$G_{p}\left(s\right)=\frac{\theta \left(s\right)}{U\left(s\right)}=\frac{K_{0}}{s\cdot \left(\tau s+1\right)}.$$

The coefficients in this equation can be derived from the following equations, which are

(2) $$K_{0}=\frac{K_{T}\rho }{BR+K_{T}K_{B}}\;and\;\tau =\frac{JR}{BR+K_{T}K_{B}}.$$

Within this framework, each robotic joint’s angular displacement over time $\theta \left(s\right)$ is directly influenced by an input control signal U, which itself is a product of data collated from sensor fusion techniques to be elaborated upon in subsequent discussions. The research approach diverged from individual parameterization; instead, it adopted a collective method where representative coefficients $K_{0}$ and τ were determined through empirical analysis derived from step response experiments. 

Figure 5b illustrates a typical block diagram of a single joint, where J represents the effective rotational moment inertia exerted on the DC motor, B is the viscos friction coefficient, R denotes the motor armature resistance, ρ signifies the gear ratio between the DC motor and the physical joint, and $K_{T}$ and $K_{B}$ represent the motor torque constant and motor back-EMF constant, respectively.

The coefficients of the involved joints were identified experimentally on an individual basis, which can be derived from previous studies [24,25]. These coefficients were used to synthesize and model the robotic platform. However, after the platform was constructed, these coefficients could change due to mechanical coupling with the attached mechanisms. Additionally, when external loads were applied, some coefficients, such as moments of inertia, deviated from their original values. Consequently, the model needed to be dynamically adjusted to ensure precise tracking performance.

### 3.3. Sensor Fusion of Position Measurements with the Kalman Filter and Complementary Filter

The design for the robotic platform’s feedback mechanism emphasized compactness and cost-efficiency by integrating accelerometer and gyroscope sensors within MPU6050 modules installed at each limb. To enhance measurement precision, sensor fusion was applied, combining inputs from both sensors to leverage the unique attributes of gyroscopes for detecting swift orientation changes and accelerometers for maintaining accuracy over extended periods. Gyroscopes tend to suffer from drift effects, compromising precision, while accelerometers are less adept at capturing high-speed maneuvers and can be prone to linear acceleration distortions. However, the synergy through sensor fusion ensured more reliable and consistent angular position readings. This section evaluates two leading sensor fusion algorithms: the Kalman filter and the complementary filter. 

The Kalman filter achieves this through a two-step process: prediction and correction.

- Prediction: The filter projects the current state estimate forward in time, accounting for uncertainties in both the system state and the measurements. This allows for a more accurate prediction compared to relying solely on a single measurement.
- Correction: Once the prediction is made, actual sensor measurements are used to refine the state estimate and its associated uncertainty. The filter calculates a weighted average, where the weights are determined by the Kalman gain. This gain reflects the confidence placed in the new measurement based on sensor reliability.

**Figure 6:**
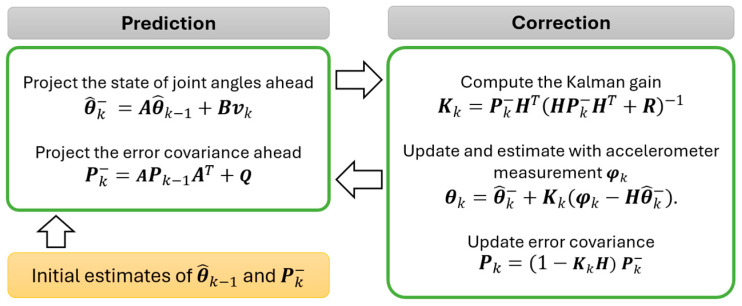
Procedures of sensor fusion utilizing the Kalman filter algorithm, demonstrating the sequential steps for combining accelerometer and gyroscope measurements to estimate pitch angles with reduced noise and enhanced accuracy.

This continuous update process allows the Kalman filter to learn and improve its estimates with each new data point. The core strength of the Kalman filter lies in its ability to statistically account for both process and measurement noise. This enables it to deliver high-fidelity estimations, making it a valuable tool for applications requiring precise motion detection and control. In this application, the algorithm combined data from gyroscopes and accelerometers, commonly used for motion tracking. Figure 6 shows the updating process of the Kalman filter. By effectively integrating measurements from these various sensors, the Kalman filter significantly enhances the reliability and accuracy of the sensor fusion process.

In this prediction step, the matrix H relates the current state variables to the measurement, Q represents the process noise covariance, and R is the measurement noise. As for the variables, ${\hat{\theta }}_{k}^{-}$ represents the posteriori state variables, $v_{k}$ denotes the control input, $P_{k}$ signifies the error covariance, $K_{k}$ is the Kalman filter gain, and $\varphi_{k}$ is the pitch angle measured by the accelerometer. It is assumed that the system state of the sensors does not change between two steps, so the control input $u_{k}$ can be zero. In this study, various combinations of R and Q values were experimentally tested to optimize the sensor performance. Specifically, the measurement noise R, associated with the accelerometer, was set to 5 to achieve the optimal results. The process noise covariance Q, determined through trial and error, was set at a constant value of 0.1. The state variables were adjusted to integrate measurements from both the gyroscope and accelerometer, allowing the Kalman filter to be set up accordingly. 

In the Kalman filter algorithm, the parameters included the predicted angle, denoted as $\theta_{1,k}$, and the targeted gyro angle, denoted as $\theta_{2,k}$. The state vector ${\hat{\theta }}_{k}$ is a 2 × 1 vector ${\left[\begin{array}{cc} {\hat{\theta }}_{1,k} & {\hat{\theta }}_{2,k} \end{array}\right]}^{T}$. Based on the calibration of sensors, the coefficients of state matrix A were determined, which led to the following equation:

(3) $${\hat{\theta }}_{k}^{-}=\left[\begin{array}{c} {\hat{\theta }}_{1,k}^{-}\\ {\hat{\theta }}_{2,k}^{-} \end{array}\right]=\left[\begin{array}{cc} 1 & a_{12}\\ 0 & 1 \end{array}\right]\cdot \left[\begin{array}{c} \theta_{1,k}\\ \theta_{2,k} \end{array}\right].$$

If the error covariance $P_{k}$ was defined as

(4) $$P_{k}={\left[\begin{array}{cc} P_{00} & P_{01}\\ P_{10} & P_{11} \end{array}\right]}_{k},$$

the matrix was updated itself by:

(5) $$P_{k}^{-}=\left[\begin{array}{cc} 1 & a_{12}\\ 0 & 1 \end{array}\right]\cdot P_{k-1}\cdot \left[\begin{array}{cc} 1 & 0\\ a_{12} & 1 \end{array}\right]+\left[\begin{array}{cc} Q & 0\\ 0 & Q \end{array}\right]\Delta t.$$

With this information, the Kalman filter gain, $K_{k}$, represented as ${\left[\begin{array}{cc} K_{0,k} & K_{1,k} \end{array}\right]}^{T}$, can be updated using the following equation.

(6) $$K_{k}=P_{k}^{-}\cdot \left[\begin{array}{c} 1\\ 0 \end{array}\right]\cdot {\left(\left[\begin{array}{cc} 1 & 0 \end{array}\right]\cdot P_{k}^{-}\cdot \left[\begin{array}{c} 1\\ 0 \end{array}\right]+R\right)}^{-1}.$$

Additionally, since the relationship between current state and measurement was one-to-one conversion, the vector of H becomes ${\left[\begin{array}{cc} 1 & 0 \end{array}\right]}^{T}$. Thus, the predicted angle $\theta_{1,k}$ was calculated as

(7) $$\theta_{1,k}={\hat{\theta }}_{1,k}^{-}+K_{0,k}\cdot (\varphi_{1,k}-{\hat{\theta }}_{1,k}^{-}).$$

In this equation, $\varphi_{1,k}$ represents the angle measured by the accelerometer, and the error covariance can be updated using a similar approach. 

The Kalman filter is a technique designed to estimate the state of a dynamic system by processing a series of noisy and incomplete data points. On the other hand, the complementary filter offers a simpler alternative. Its core principle is to merge and adjust the proportions of the two signals obtained from the sensors to achieve an improved result. This principle is applied through the following algorithm.

(8) $$\theta_{1,k}=\alpha \cdot \theta_{2,k}+\left(1-\alpha \right)\cdot \varphi_{k}.$$

In this equation, the coefficient α is constrained to be between 0 and 1. While the Kalman filter can yield precise joint angle estimates, it requires more computational steps. Conversely, the complimentary filter achieves comparable estimates with fewer calculations. Despite the accurate results calculated by the Kalman filter, minor oscillations caused by uncompensated noises can still occur, potentially leading to unwanted vibrations in the driving DC motors. Due to these considerations, the complementary filter algorithm was chosen and used in the experimental platform.

### 3.4. Nonlinear Factors of Physical Setup

The robotic platform utilized in this study was influenced by various nonlinear factors impacting its operational dynamics. These factors within a single joint include the following: (1) motor dead-zones; (2) inconsistent friction coefficients; (3) varying inertia based on joint positions; (4) backlash in gear mechanisms; and (5) elastic deformations in the twisted-string actuators.

Among these, the dead zones in the DC motors and the saturation of the power supply’s output voltage were particularly significant. The joints demonstrated dead zones where the motors would not respond to control commands if the command voltage was within ±0.6 V. To address this, control signals below this threshold were boosted to at least 0.6 V to initiate motor movement. Additionally, power saturation occurred when the control commands generated by the controller approached the voltage limits of the power supply, leading to potential clamping of the output. Moreover, loading effects could arise if the required current exceeded the capacity of the power amplifiers, especially under sudden torque demands or excessive loading. To protect the DC motors, the control commands were restricted to a maximum of ±8 V, with a current limit of 1 A per motor. 

Inconsistent friction was noticeable when the motor rotated in opposite directions and fluctuated slightly with changes in the load applied to the joint. Although this friction could be estimated to some extent, variations persisted during actual operation, with different joints exhibiting differing friction levels. This inconsistency made it difficult to compensate accurately using a linear controller. As a result, an adaptive mechanism was employed to approximate the friction’s magnitude in the controller and effectively compensate for the angular movement assigned to each specific joint.

Mechanical components could also introduce nonlinearities into the system. Minor misalignments and backlash of transmission created during manufacturing can disrupt smooth operation. As the posture of the robot platform changes, the effective moments of inertia, J, of both upper and lower limb joints changed continuously. Even with lubrication, some friction remained, acting as a constant external disturbance. Additionally, slight imbalances or eccentricities of joints during rotation could lead to periodic changes in both angular speed and position. The frequency of these disturbances varied with the rotational velocity of the joint, requiring adaptive control strategies to minimize their impact on overall system performance. To address these nonlinear issues, the controller for each joint was designed with these nonlinearities explicitly considered.

These nonlinear factors were identified through experimental analysis, during which the behavior of the robotic platform was meticulously observed under various operating conditions. The parameters and ranges of these nonlinear factors, such as dead zones, inconsistent friction, and changing inertia, were derived by systematically varying input commands, loads, and joint positions and measuring the platform’s response. These data were then analyzed to establish the specific thresholds and ranges of the nonlinearities affecting each joint. Once these ranges were determined, they were integrated into the simulation model of the robotic platform to ensure an accurate representation of its real-world dynamics. Additionally, tailored compensation strategies were developed and implemented within the control system to mitigate these nonlinear effects, ensuring more stable and precise operation across different scenarios.

## 4. Identification of Human Motions and Path Planning

### 4.1. Reference Trajectories of Selected Motions

The primary goal of this experimental robotic platform was programmed to replicate specific tasks performed by the user, focusing on the movement of the right arm. The robot’s joint positions were determined by angular trajectories for the elbow and shoulder joints. As mentioned in the previous section, these reference trajectories were derived from pre-derived human reference motions captured through multiple repetitive motion recordings by a test subject. Each trajectory detailed the angular movement and range of the selected joint. Timing for these trajectories was expressed as a percentage of the overall cycle rather than in seconds, allowing for adjustments based on the physical scenario. This required modifying the initial and end positions, timing, and magnitude of the motions to fit the specific application. The motions of the right arm were emphasized because it played a major role in the tasks studied, ensuring detailed and accurate movements that mimic those of the user’s dominant arm [13].

To compensate for the joint movements of the robotic platform, it was essential to synthesize reference trajectories of selected motions for the joints involved in specific motions. This process collected sufficient motion data to extract featured characteristics. While motion prediction models using CNNs could effectively identify motions, they fell short in synthesizing the required trajectories due to a lack of precise time information. Even when time information was included, it remained insufficient for creating complete trajectories due to variations in the duration of each motion. Consequently, reference trajectories for the involved joints must be derived from the recorded movements of the same motion, repeatedly performed by the same user or test subject. Instead of using angle-to-angle information, time series of joint movements must be employed to synthesize the trajectories of reference movements. However, since the reference movements derived from pre-recorded data were unlikely to match the duration of the ongoing movement, the trajectories of the involved joints needed to be adjusted based on the acquired ongoing trajectories. This adjustment ensured that the robotic platform can accurately replicate the intended motions, despite variations in individual motion durations and initial positions.

To synthesize reference trajectories for various movements, it was necessary to collect a sufficient number of motion samples. After collecting these samples, a temporal alignment method, dynamic time warping (DTW), was employed to align the collected trajectories. The initial step involved aligning the collected motion samples to a common timeframe, facilitating the comparison among the acquired motions and characteristic extraction for the reference motions. DTW, a widely used tool for temporal alignment, minimized the effects of time shifts and distortions by creating a warping path that detected similar characteristics between signals at different phases [26]. This method adopted a nearest neighbor algorithm with DTW as the distance measure, demonstrating a high level of effectiveness in identifying motion profiles within multivariate series. By aligning the collected data onto a common timeframe, DTW enabled the generation of accurate and consistent reference trajectories from the motion samples.

To align joint trajectories from two recorded motions, individually acquired angular positions presented as two time series, X and Y, and were expressed as

(9) $$X=(x_{1},x_{2},\ldots ,x_{T_{1}}),\;Y=(y_{1},y_{2},\ldots ,y_{T_{2}}).$$

The two recorded trajectories X and Y have distinct time durations, which are $T_{1}$ and $T_{2}$. Calculating a distance metric between two vectors representing the same position is challenging if the datasets are not aligned to the same scale. Therefore, a local cost measure is defined as follows:

(10) d:F×F→R≥0,

To determine the best similarity between two recorded datasets presented as distinct time sequences, a cost measure was used. This measure evaluated similarity, with a lower cost indicating more similar characteristics and a higher cost indicating less similarity. The cost was calculated locally as a matrix for each component in the matrix, measuring distances to the four surrounding components. The process began at the initial boundary $\left(x_{1},{\;y}_{1}\right)$ and continued to the end boundary $\left(x_{T_{1}},y_{T_{2}}\right)$. Due to different durations of the two separate time series, $T_{1}$ and $T_{2}$, the recorded trajectories X and Y did not have direct one-to-one mapping. The cost sought the minimal distance values at each step, storing these costs in a mapping. Once the calculation was complete, the two recorded trajectories could be aligned to the same timeframe. This alignment process standardized the motions onto a uniform timeframe, represented on a percentage scale from 0% to 100%. By converting all sampled data to this consistent timeframe, it became possible to extract motion characteristics for synthesizing a reference trajectory. This method ensured precise alignment and comparison of the recorded datasets.

**Figure 7:**
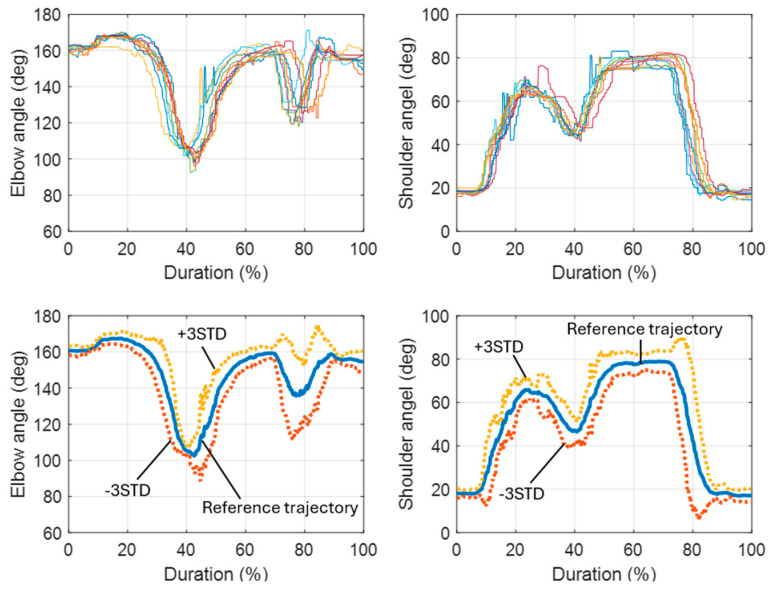
Motion profiles adjusted through temporal alignment and the derived reference trajectories for the elbow and shoulder joints (object lifting). The top two figures display ten recorded motions aligned from 0 to 100%, while the lower figures present the derived reference trajectories for the motion.

This method was employed to create reference trajectories for selected motions. When a motion is detected by the motion prediction model, the subsequent trajectories of the involved joints can be retrieved from these established reference trajectories. Figure 7 depicts the outcomes of processing 10 recorded motions involving elbow and shoulder joints (object lifting) using DTW. Originally, the durations of these motions ranged from 5 to 7.5 s. The horizontal axis represents relative time scaled from 0% to 100%, rather than absolute seconds. The average profiles extracted from individual motions were used as their respective reference trajectories. Additionally, Figure 7 displays the average motion trajectories from 30 samples, with boundaries defined at ±3 standard deviations.

### 4.2. Reference Motions

**Figure 8:**
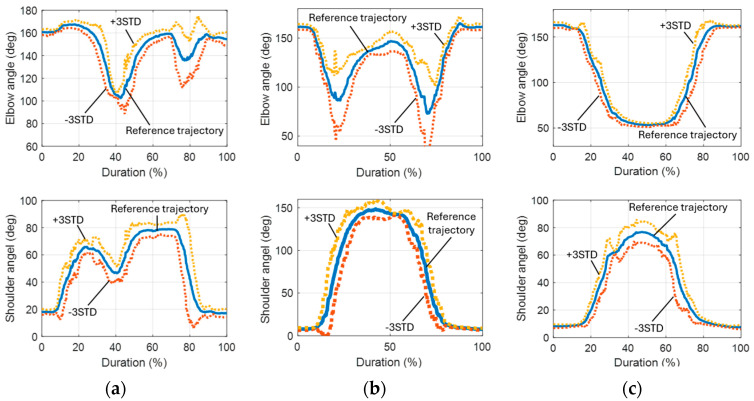
Derived reference trajectories of three selected motions: (**a**) object lifting, (**b**) raising the right arm, and (**c**) drinking.

The three reference motions selected for this study were (1) object lifting, (2) raising the right arm, and (3) drinking. The trajectories for these reference motions, including both elbow and shoulder joints, are illustrated in Figure 8. These trajectories were adjusted and extended based on the parameters obtained from the detected motions.

### 4.3. Adjustments of Reference Trajectories

Once the reference trajectories of selected motions are established, path planning for the robotic platform’s joints can be synthesized according to the real movement acquired from the user. However, as discussed in a preceding section, reliable motion prediction is only feasible after completing at least one-third of the motion. It was observed that arm movements varied from time to time, requiring adjustments to the real trajectories based on the actual situation. While the motion profile remained consistent, deviations could occur due to various factors, such as inconsistent amplitude, drifting initial position, varying total motion duration, and collision with external objects. To accommodate these factors and ensure accurate execution by the robotic platform, adjustments to the reference trajectories of the predicted motions were necessary.

**Figure 9:**
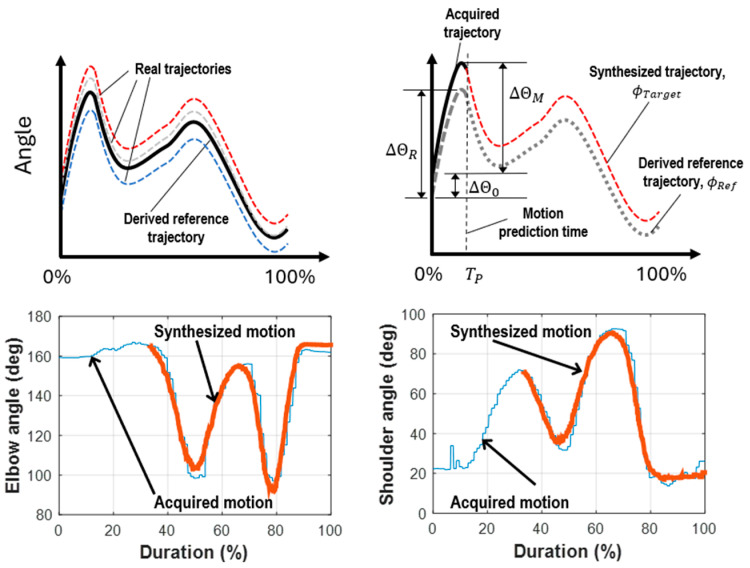
Adjustment of joint trajectory based on reference motions and real-world movements.

To address these factors, adjustments to the reference trajectories were necessary. The initial positions of involved joints should be aligned to the same starting angular positions to ensure that the robot can generate a similar motion. Joint movement amplitudes were scaled based on data from the first one-third of the predicted motion. This specific time is defined as the motion prediction time, $T_{P}$. Additionally, the total duration of the motion was adjusted according to the duration of this one-third motion cycle. Figure 9 illustrates the process of generating synthesized trajectories. Initially, real trajectories of a specific motion were consolidated into a reference trajectory. This reference trajectory was then adjusted based on the actual acquired motion using several required parameters. Once these adjustments were made, minor refinements were applied to ensure that the generated trajectories maintained identical angular positions at both the starting time and $T_{P}$ when the trajectories were transferred to the robotic platform.

The synthesized trajectory was adjusted from the reference trajectory based on the actual movement scenarios. Two major adjustments were required: (1) the deviation in the start position between the real and reference trajectories, denoted as $∆\Theta_{0}$, and (2) the adjusting ratio, $∆\Theta_{M}/∆\Theta_{R}$, which scaled the amplitude of the reference trajectory based on the comparison between the real and reference trajectories. The amplitudes $∆\Theta_{R}$ and $∆\Theta_{M}$ represented critical variations used to determine the scaling between the two trajectories by comparing the peak variation of the joint angular position with the starting angular position. Accurately determining whether the robot’s operator had driven the platform to the targeted duration $T_{P}$ can be challenging, so both angular velocity and angular position were measured to ensure precise adjustments.

The adjustment process began with collecting necessary motion data to select reference trajectories for involved joints. Initial angular positions of the joints were recorded to identify any initial deviations. The user’s angular positions and angular velocities were acquired and assessed. When the motion reached the motion prediction time, $T_{P}$, which was set to be one-third, the prediction model determined the type of the ongoing motion. After determining the motion type, the reference trajectories of the predicted motion were adjusted and scaled to generate the targeted trajectories of the joints. The angles of the targeted trajectories, $\phi_{Target}$, for a single joint were generated using the corresponding angular position of the reference trajectory, $\phi_{Ref}$, an adjusting ratio of amplitude $∆\Theta_{M}/∆\Theta_{R}$, and the shifted angle $∆\Theta_{0}$. Thus, a simple linear approximation could be applied to obtain the targeted trajectories for individual joints, expressed as

(11) $$\phi_{Target}=\frac{∆\Theta_{M}}{∆\Theta_{R}}\cdot \phi_{Ref}+∆\Theta_{0}.$$

These targeted trajectories of individual joints can then be applied to the controllers to drive the robotic platform for specific motions. Figure 9 illustrates the synthesized results of the planned paths for the object lifting motion. Observations show that the synthesized path closely matched the real motion profile. However, deviations of up to 20° at the elbow joint and 16° at the shoulder joint were noted. These deviations might be caused by vibration or inconsistency in the subject’s arm movements. Further, psychophysical analysis was necessary to determine whether these deviations can be disregarded.

### 4.4. Scalability of Acquired Reference Trajectories

Building on prior research, it has been observed that the selected motions exhibit both self-similarity and consistency across different individuals [13]. To make these reference trajectories applicable to a broader population, the trajectories can be scaled according to factors such as body size, total duration, and other physical characteristics. By adjusting these parameters, the acquired reference trajectories can be modified—either expanded or contracted—to meet the specific needs of different users and various situations.

The ability to scale these reference trajectories within the reasonable limits of human movement ensures their adaptability. However, extending these trajectories to include unstudied motions presents significant challenges. This would require gathering additional data on various movements, potentially involving the segmentation of individual motions into smaller components. These segments could then be reassembled to form a complete motion. While machine learning techniques can assist in this reassembly process, certain actions, particularly those influenced by cultural practices, professional requirements, or personal habits (e.g., dining or dressing), cannot be easily reconstructed from a motion library. The process of segmentation and reassembly is complex and represents a separate area of research beyond the scope of this study.

The modeling and path planning approach proposed in this study could potentially be applied to other motions, provided the training procedures and sequences are standardized and streamlined for predicting individual motions. However, it is important to note that the model is best suited for self-similar motions. Motions that do not exhibit consistency over time are not well-suited for this path planning approach. Future research might explore the feasibility of segmenting motions into smaller components, but this would likely require a redesigned learning and path planning methodology.

## 5. Controller Synthesis and Tracking Performances

To ensure that the angular movements of the robotic platform were compensated properly, both a linear controller and a nonlinear controller were designed for motion tracking. These controllers were planned to be implemented using an Arduino mega 2560 MCU, operating at a sample rate of 100 Hz. The controllers were developed based on the nominal dynamics of individual actuators, while the cross-coupled dynamics [27,28] were treated as external disturbances as well as the unsynchronized deviations of individual joints.

### 5.1. Synthesis of Linear Controller and Adaptive Robust Controller

A linear controller for motion tracking of a single joint was designed using the pole-placement method, based on the characteristic equation identified earlier. Its tracking performance was compared to that of a unity feedback controller. Given that the robotic platform operates both the upper and lower arms simultaneously, cross-coupled dynamics can act as external disturbances. Therefore, an important consideration is that the synthesized controller must effectively attenuate disturbances and operate within the linear region of the power source without saturation.

**Figure 10:**
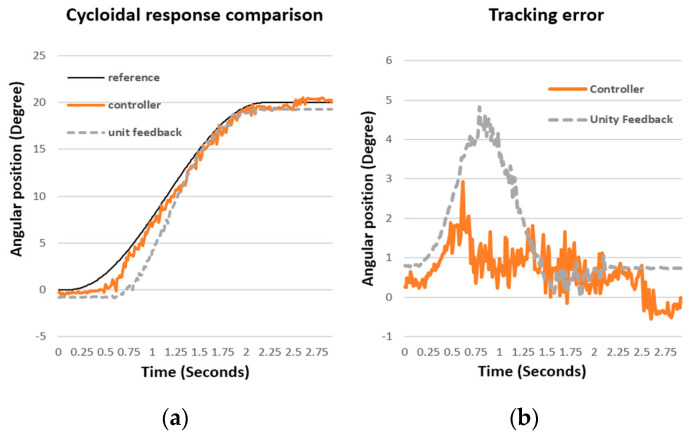
(**a**) Comparison of system response between compensated system and unity feedback control result for with and without an appropriate controller and (**b**) comparison of tracking errors of both control schemes [24].

To evaluate the performance of the synthesized controller, a cycloidal input trajectory was used as the reference trajectory for both the unity feedback system and the compensated system with the pole-placement controller. The comparison of their controlled results is illustrated in Figure 10, showing the transient response of an elbow joint. The comparison clearly demonstrates that the pole-placement controller achieves a better transient response than the unity feedback compensation, with less tracking error. The steady-state error of the compensated result is less than 5% of the total stroke, indicating superior performance of the pole-placement controller.

However, the linear controller can only compensate for the linear response of the robotic platform. The nonlinear phenomena present on the platform cannot be adequately compensated for, resulting in inaccurate position tracking. To address these issues, an adaptive robust control (ARC) scheme was implemented. This ARC scheme was specifically designed to handle identified nonlinear factors, including cross-coupled dynamics, dead zones of individual actuators, and power source saturation. The ARC controller works by continuously adapting to changes and disturbances in the system, ensuring robust performance even in the presence of nonlinearities. Cross-coupled dynamics refer to the interactions between different joints or parts of the robotic system, which can affect each other’s movements. Dead zones in actuators are regions where small control signals do not produce any movement, leading to delays and inaccuracies. Power source saturation occurs when the power supply cannot provide sufficient voltage or current, limiting the actuator’s performance. The derivation of the rules for ARC controllers designed to enhance both tracking and synchronization accuracy in multiaxial mechanisms is detailed in [12]. 

Previous studies have explored various methods to compare the performance of different control strategies, such as linear controllers, adaptive controllers, LQR, and ARC [24,29]. These comparisons often focus on metrics like tracking accuracy, system stability, and responsiveness to disturbances. ARC, for instance, has shown superior performance in compensating for nonlinearities and uncertainties in complex systems, whereas linear controllers are typically more effective for simpler, steady-state conditions. LQR, with its optimal control approach, has been widely used in systems requiring a balance between performance and energy efficiency. However, each method has its limitations, and the comparative studies provide valuable insights into selecting the most appropriate controller based on specific system requirements and operational conditions.

In this study, the ARC controller was carefully designed to address the various nonlinearities present in the system, including motor dead zones, inconsistent friction at the joints, varying inertia, and other unmodeled uncertainties. The dead zones in the driven motors were precisely characterized to ensure that the control system could effectively compensate for these non-responsive regions. Additionally, the friction inconsistencies at the joints, which varied with load and rotation direction, were integrated into the controller’s design. The controller also accounted for the changing inertia across different joint positions and other unstructured uncertainties that could impact system performance.

The development of the ARC controller followed established methodologies from previous research, incorporating advanced techniques to estimate and compensate for these nonlinear factors in real time. Through rigorous testing and refinement, the ARC controller demonstrated its effectiveness in mitigating the adverse effects of nonlinearities, leading to significantly improved position tracking and overall performance of the multiaxial robotic system.

### 5.2. Cross-Coupled Dynamics among Joints

One important issue in compensating for the angular positions of the arm mechanism is the coupling of individual dynamics among joints. Cross-coupled dynamics can arise from various factors, such as the continuously changing inertia caused due to the varying poses of the arm, different loads held by the end effector, and vibrations induced by the mechanism’s movements. These cross-coupled dynamics are challenging to model and were typically treated as external disturbances. However, compensating for cross-coupled dynamics individually without adequate synchronization can result in inaccurate positioning.

To address this issue, the cross-coupling can be compensated for by considering the asynchronization of the performed motion. This means that the controller needs to consider the angular movements of involved joints as a whole. Deviations in one joint can affect the positional accuracy of the other joints. Therefore, the cross-coupled dynamics adopted in this platform were addressed by focusing on the synchronization error corresponding to individual joints.

### 5.3. Gain Scheduling

Experimental results from a previous study [12] demonstrate that the ARC controller can effectively compensate for several nonlinear factors, such as the dead zones when initiating movement from a stop and on-the-fly calibration of uncertain system dynamics. Furthermore, the ARC controller can prevent the actuator from operating in the saturated zone. Despite these advantages, the adaptive mechanism of the ARC controller still introduced observable oscillations. In contrast, the linear controller produced smoother tracking results during steady-state operation. However, the designed linear controller also exhibited larger tracking deviation when the control commands fell within the dead zone. 

In this study, the gain scheduling control strategy was employed to simulate the compensation for the proposed robotic platform. To ensure accurate tracking of the robotic platform, combining the strengths of both ARC and linear controllers was a feasible approach, which can be implemented through gain scheduling based on the operating conditions of each joint. The situational rules used were as follows:

- Linear Controller: Applied when the required angular velocity is less than 0.35 rad/s, and the required angular acceleration is less than 2.6 rad/s^2^.
- ARC Controller: Applied in all other situations.

These rules were used to compensate for both the elbow and shoulder joints of the robotic platform. Such an approach ensured fast response time during transient states as well as smooth tracking performance during steady states. 

### 5.4. Simulation Results

**Figure 11:**
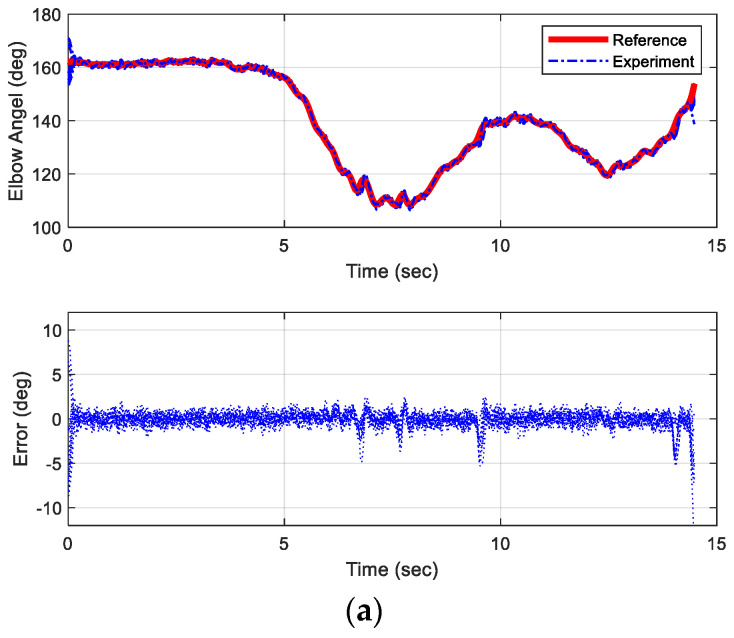

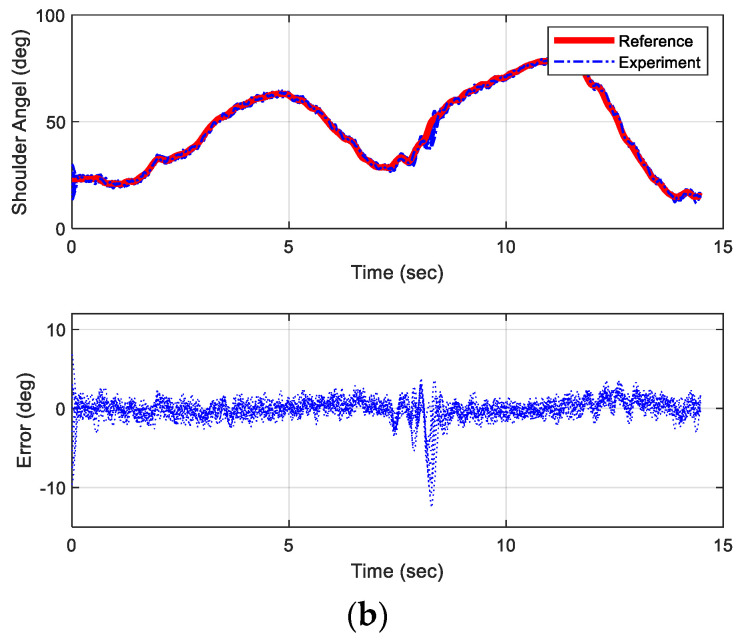
Ten trials of compensated results of elbow and shoulder joint movements (object lifting). (**a**) Tracking performance and tracking error of elbow joint and (**b**) tracking performance and tracking error of shoulder joint.

To validate the effectiveness and repeatability of the proposed controllers, the trajectories for the task of object lifting were used as the reference motion for the robotic platform. The original duration of this motion ranged from 6 to 7.5 s for the test subject. For testing purposes, this reference motion was expanded to 15 s to evaluate the tracking performance of the controller. Given the reference motion trajectories, Figure 10 illustrates the simulated tracking performance and corresponding errors of both elbow and shoulder joints for an object-lifting motion. The reference motion for this experiment was obtained from a single test subject. The controllers, based on gain scheduling and the situational rules, demonstrated superior tracking errors, maintaining errors within 5% of the total angular spans for both joints. However, the angular accelerations and velocities of the actuators at the involved joints were constrained by the maximum voltage limits of the DC motors. Consequently, the profiles of generated forces might not perfectly match the profiles of the original motion. If the required speeds or accelerations exceed these limits, delays and fluctuations might still occur while tracking the reference trajectories. To evaluate the repeatability of the proposed control strategy, Figure 11 illustrates the error distribution across 10 simulated trials. In these simulations, the initial angular positions of both elbow and shoulder joints were randomly set to vary within a deviation of 10°. Without significant external disturbance, the majority of the errors in the involved joints were confined within a 12° range. It is evident that the hardware setup exhibited some physical defects at specific angular positions of the shoulder joint.

## 6. Operation of Proposed Robotic Platform

### 6.1. Operational Process of the Robotic Platform

The operational sequence of the proposed robotic platform began with motion acquisition using a depth camera. Once the movements of individual joints were recorded, the angular positions of these joints were fed into a motion prediction model to determine the type of ongoing motion. This model utilized a pre-built motion library that contained reference trajectories for various motions.

**Figure 12:**
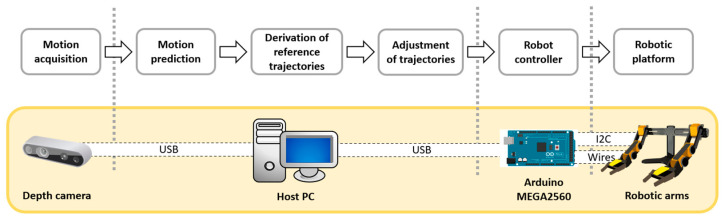
Operational process of the robotic platform to mimic human arm motions.

From the motion library, the system derived the angular trajectories for the involved joints. These reference trajectories were then adjusted based on the acquired motion data from the user, taking into account factors such as amplitude, shifted angles, and the total duration of the motion. This adjustment ensures that the robotic platform can accurately replicate the intended human motions. The adjusted trajectories were subsequently sent to the controllers of the robotic platform. These controllers, which integrated both ARC and linear control strategies through gain scheduling, used the adjusted trajectories to compensate and drive the joints of the robotic platform. The dual control strategy ensures both fast response times during transient states and smooth tracking performance during steady states. Figure 12 illustrates the overall process for a complete cycle of the robot’s operations to mimic human arm motions, from initial motion acquisition to the execution of the adjusted trajectories by the robotic platform. This comprehensive approach ensures precise and effective replication of human motions by the robotic system. In this study, the compensated trajectories of the selected motions were simulated.

### 6.2. Response Time for Adopted MCU

While the control strategy effectively followed the motion when the duration was sufficiently long, it is important to identify the types of motions that the robotic platform can reliably mimic. Given that the time constant of the motors at individual joints ranged from 0.4 to 0.6 s, the platform must be selective about the motions it attempts to replicate to ensure accuracy and responsiveness.

To evaluate the feasible motion cycles that the robot can follow, two specific motions were simulated: object lifting and hand raising with the right arm. These motions were chosen because they are typical and straightforward actions that involve significant joint movements. Normally, these motions can be completed within approximately 6 to 7.5 s. For the purposes of this evaluation, the trajectories for these motions were rearranged to vary between 5 and 15 s while maintaining an identical sample rate. This range allows for the assessment of the platform’s performance across different speeds and durations, providing insight into its capability to mimic human motions accurately. By testing these adjusted trajectories, we can determine the limits and optimal conditions for the robotic platform’s operation in real-world scenarios.

**Figure 13:**
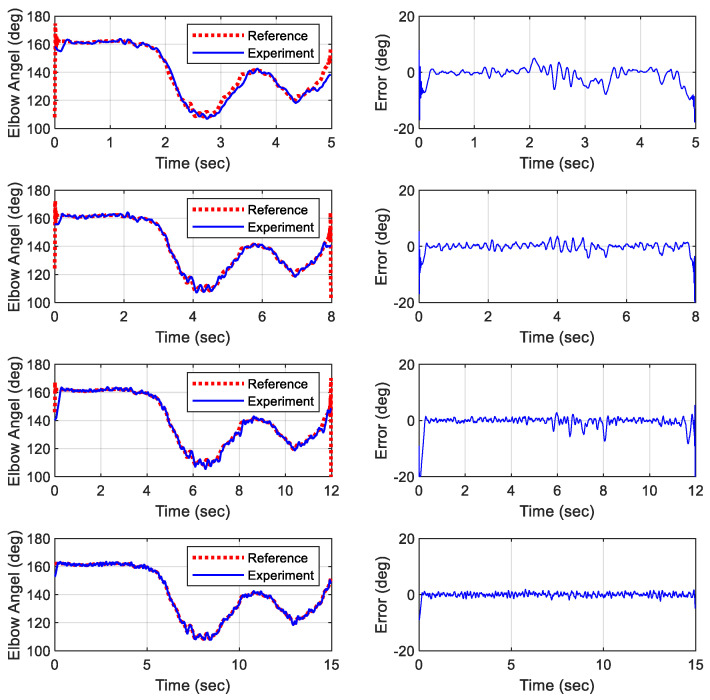
Simulated results of elbow joint with different operation durations for object lifting motion.

**Figure 14:**
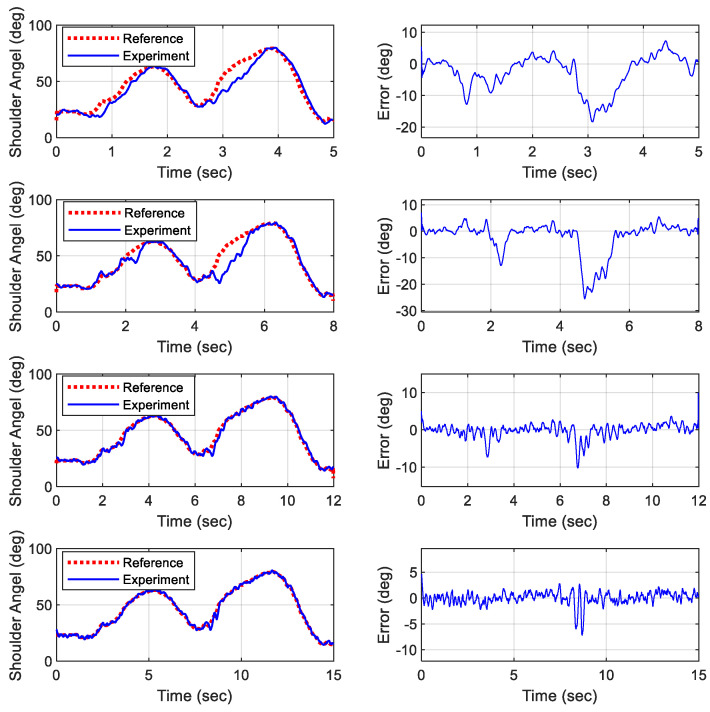
Simulated results of shoulder joint with different operation durations for object lifting motion.

Figure 13 and Figure 14 display the simulated results of the object lifting motion completed over various durations. The simulations indicated that the deviation between the reference trajectories and the physical outputs for both the elbow and shoulder joints exceeded 10° when the total duration of the motion was less than 8 s. If the duration is reduced below a threshold, the deviation can increase significantly. This discrepancy is likely due to the slow time constants of the actuators used. Therefore, it is recommended that the total duration of a motion for this adopted robotic platform should not be shorter than the original duration to avoid significant deviations due to the limited response time of the actuators.

Extending the duration of the motion can offer several advantages. It allows for smoother and more controlled movements, reducing the risk of mechanical stress and wear on the robotic components. Additionally, a slower, more deliberate motion can improve the precision of the robot’s actions, making it more suitable for tasks that require fine motor skills and careful handling. On the other hand, performing motions faster than a threshold can lead to several issues. The primary concern is the potential for increased deviations due to the actuators’ inability to respond quickly enough. This can result in inaccurate positioning and compromised performance. For the proposed robotic platform, it is recommended to extend the motion cycle by one-third beyond its original duration to ensure accurate tracking. Moreover, rapid movements can introduce additional mechanical stress and potential instability, which might lead to premature wear or damage to the robotic components. Thus, while it is advantageous to extend the motion duration for certain applications, care must be taken not to reduce the duration below the original timeframe to maintain accuracy and protect the mechanical integrity of the robotic platform.

### 6.3. Experimental Results of Proposed Gain Scheduling Controller

**Figure 15:**
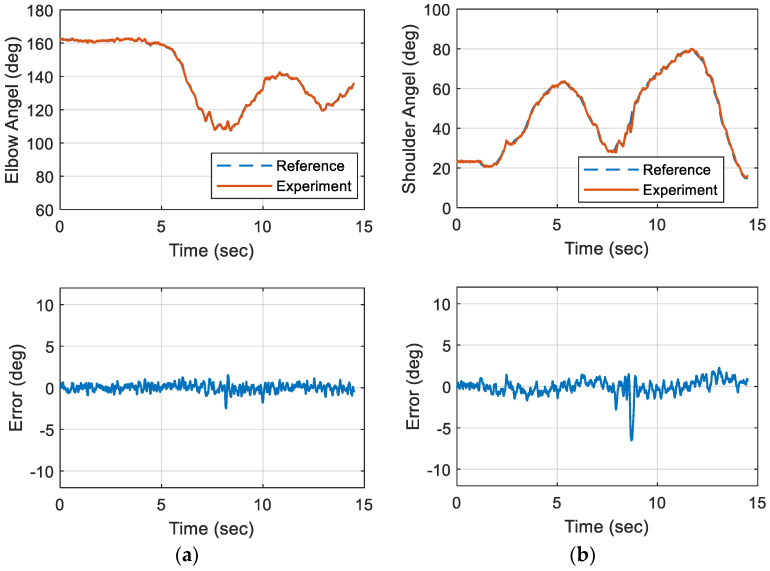
Compensated results of elbow and shoulder joint movements (object lifting). (**a**) Tracking performance and tracking error of elbow joint and (**b**) tracking performance and tracking error of shoulder joint.

The object-lifting motion was used as the reference trajectory for the robotic platform. Figure 15 displays the tracking performance and corresponding errors for both the elbow and shoulder joints of this selected motion over a 15 s period. With the implementation of the proposed controller and operational rules, tracking errors were maintained within 5% of the total angular spans. However, the angular accelerations and velocities of the actuators at the involved joints were constrained by the maximum voltages that could be applied to the DC motors. When the required speeds or accelerations exceeded these limits, delays and fluctuations were observed in tracking the reference trajectories.

**Figure 16:**
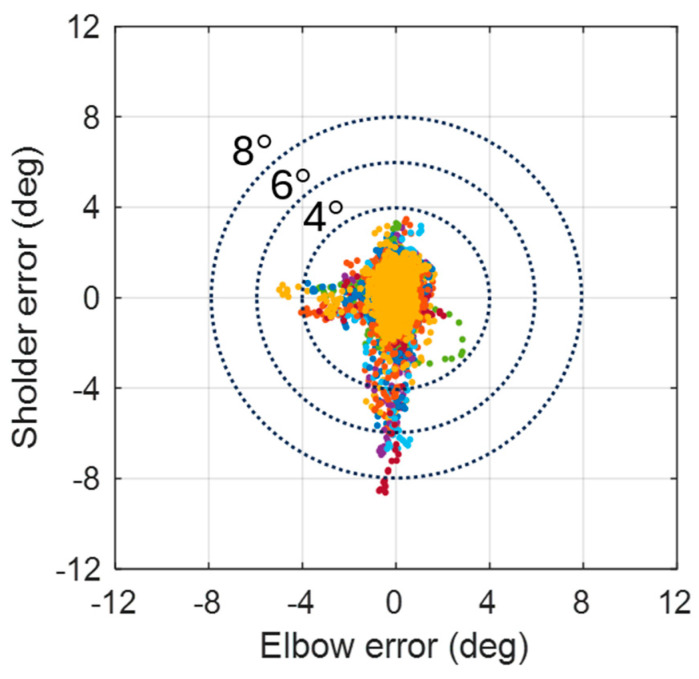
Distribution of tracking errors of the selected motion (10 trials of object lifting).

To evaluate the repeatability of the proposed control strategy, Figure 16 illustrates the errors distribution over 11 trials of experimental results. Most errors of involved joints were confined within the ±8° range, with over half falling within ±4°. When the ARC controller was integrated with the linear controller, tracking errors were significantly reduced. This indicated that combining both nonlinear and linear controllers resulted in optimal tracking performance.

## 7. Conclusions

In this study, an integrated control strategy combining both ARC and linear controllers has been developed to address reference tracking and nonlinear factors in a robotic platform. The proposed controller demonstrated adequate tracking performance for the multiaxial robotic system, effectively managing the complexities of motion recognition and trajectory planning. The motion recognition part, which allows CNNs to process angle-to-angle representations of joint movements, proved essential for predicting and replicating human-like motions in real-time. This integration enables the robotic platform to assist human workers by accurately mimicking their movements.

However, the complexity of implementing this controller on an MCU unit remains a challenge. Additionally, the cross-coupled dynamics (CCD) between the two axes were not considered, which is a crucial factor when the robot interacts with human users. In future work, more joints will be integrated into the control strategy, and the perturbations caused by CCD will be thoroughly investigated. Efforts will also be made to simplify the implementation of the controller on the selected MCU unit to achieve a faster sample rate. This will enhance the system’s responsiveness and reliability in dynamic, real-world environments.

## Figures and Tables

**Table 1:** Estimated results of motions with different targeted duration (100%, 50%, 33%, and 20%) [13].

Type of Motion	Drinking	Arm Raising	Object Lifting
Cycle (%)			
100%	100%	100%	100%
50%	100%	100%	100%
33%	97.14%	100%	97.14%
20%	~35%	~35%	~35%

## Data Availability

The data that support the findings of this study are available from the corresponding authors upon reasonable request.
